# The Bony Resection Accuracy with Patient-Specific Instruments during Total Knee Arthroplasty: A Retrospective Case Series Study

**DOI:** 10.1155/2021/8674847

**Published:** 2021-02-15

**Authors:** Liang Yuan, Bin Yang, Xiaohua Wang, Bin Sun, Ke Zhang, Yichen Yan, Jie Liu, Jie Yao

**Affiliations:** ^1^Department of Orthopedics, Peking University International Hospital, Beijing 102206, China; ^2^Key Laboratory for Biomechanics and Mechanobiology of Ministry of Education, School of Biological Science and Medical Engineering, Beijing Advanced Innovation Centre for Biomedical Engineering, Beihang University, Beijing 100191, China; ^3^Department of Vascular and Endovascular Surgery, Chinese PLA General Hospital, Beijing 100853, China

## Abstract

**Purpose:**

Bony resection is the primary step during total knee arthroplasty. The accuracy of bony resection was highly addressed because it was deemed to have a good relationship with mechanical line. Patient-specific instruments (PSI) were invented to copy the bony resection references from the preoperative surgical plan during a total knee arthroplasty (TKA); however, the accuracy still remains controversial. This study was aimed at finding out the accuracy of the bony resection during PSI-assisted TKA.

**Methods:**

Forty-two PSI-assisted TKAs (based on full-length leg CT images) were analyzed retrospectively. Resected bones of every patient were given a CT scan, and three-dimensional radiographs were reconstructed. The thickness of each bony resection was measured with the three-dimensional radiographs and recorded. The saw blade thickness (1.27 mm) was added to the measurements, and the results represented intraoperative bone resection thickness. A comparison between intraoperative bone resection thickness and preoperatively planned thickness was conducted. The differences were calculated, and the outliers were defined as >3 mm.

**Results:**

The distal femoral condyle had the most accurate bone cuts with the smallest difference (median, 1.0 mm at the distal medial femoral condyle and 0.8 mm at the distal lateral femoral condyle) and the least outliers (none at the distal medial femoral condyle and 1 (2.4%) at the distal lateral femoral condyle). The tibial plateau came in second (median difference, 0.8 mm at the medial tibial plateau and 1.4 mm at the lateral tibial plateau; outliers, none at the medial tibial plateau and 1 (2.6%) at the lateral tibial plateau). Regardless of whether the threshold was set to >2 mm (14 (17.9%) at the tibial plateau vs. 12 (14.6%) at the distal femoral condyle, *p* > 0.05) or >3 mm (1 (1.3%) at the tibial plateau vs. 1 (1.2%) at the distal femoral condyle, *p* > 0.05), the accuracy of tibial plateau osteotomy was similar to that of the distal femoral condyle. Osteotomy accuracy at the posterior femoral condyle and the anterior femoral condyle were the worst. Outliers were up to 6 (15.0%) at the posterior medial femoral condyle, 5 (12.2%) at the posterior lateral femoral condyle, and 6 (15.8%) at the anterior femoral condyle. The percentages of overcut and undercut tended to 50% in most parts except the lateral tibial plateau. At the lateral tibial plateau, the undercut percentage was twice that of the overcut.

**Conclusion:**

The tibial plateau and the distal femoral condyle share a similar accuracy of osteotomy with PSI. PSI have a generally good accuracy during the femur and tibia bone resection in TKA. PSI could be a kind of user-friendly tool which can simplify TKA with good accuracy. *Level of Evidence.* This is a Level IV case series with no comparison group.

## 1. Introduction

Total knee arthroplasty (TKA) has been a reliable option with excellent long-term results for patients suffering from advanced knee osteoarthritis and other severe knee diseases [1–4]. One of the keys to a good TKA is the reconstruction of the lower extremity mechanical axis. To achieve this purpose, precise bony resection and accurate prosthesis implanting are necessary. Implanting each prosthesis component as accurately as possible has always been an essential principle in TKA, and implant malalignment is considered a common cause of failure [5, 6]. To improve the accuracy of prosthesis implantation and facilitate the surgery, patient-specific instruments (PSI) were introduced to TKA [7].

PSI are usually made based on the patient's computer tomography (CT) or magnetic resonance imaging (MRI) data [8–10], which can be directly used to guide patient-specific osteotomy during TKA. Three-dimensional (3D) models of the patient's anatomy are derived and developed from CT/MRI data, and then a simulated surgery is performed on the model with some software. During this process, the parameters required for osteotomy of the knee joint would be accurately calculated. PSI are produced by 3D-printing technology according to these parameters. PSI became a favorable bridge between preoperative planning and the final TKA surgery, which allowed the surgeon to copy the surgical plan to a specific patient. Thus, its accuracy has been one of the main concerns of PSI.

3D-printed PSI have been used in TKA for more than 15 years; however, its accuracy reported by the literature was not satisfactory [11–16]. As for these results, there can be many kinds of interpretations. Delport and Vander Sloten put forward several vital points worth pondering in the letter to the Journal of Arthroplasty [17]. Inherent to this technology was that patients were treated with personalized surgical plans. PSI played a role in transferring the preoperative plans to the patients' anatomy during TKA. The intended output of PSI-assisted TKA is a postoperative result which is identical to the preoperative plan. Many researchers compared X-ray-based postoperative results with the CT/MRI-based preoperative plan regardless of the consensus amongst clinicians that a postoperative X-ray image could not qualify as an accurate measurement tool [17]. In this way, the error caused by the measurement tool might have put the blame on PSI. Therefore, we should be cautious with those results based on an X-ray study. Just as Delport and Vander Sloten concluded [17], an accurate measurement tool should be used, and the postoperative result should be compared to the actual preoperative plan.

Some researchers smartly came up with a way to study PSI accuracy without using X-ray measurement. In these studies, the amount of bony resection was measured and compared with planned osteotomy [18–22]. However, the results of these studies were covered with controversy. A study with meta-analysis has also reported controversial results [8]. The present study is focused on this issue by evaluating the efficacy of PSI in copying the bony resection reference value from the surgical plan. We assume that PSI has good accuracy in bone resection during TKA.

## 2. Materials and Methods

### 2.1. Study Population

**Figure 1:**
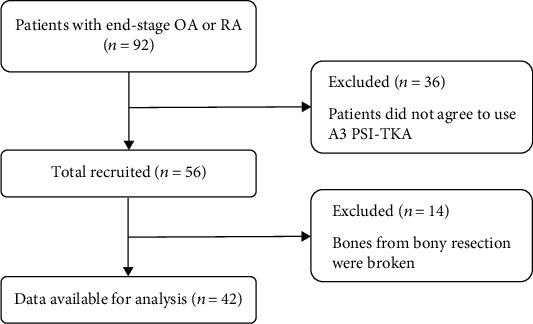
Study flowchart.

A retrospective review of all the PSI-assisted TKAs from January 2019 to June 2020 was conducted. Inclusion criteria were patients suffering end-stage osteoarthritis or rheumatoid arthritis of the knee without operational contraindications and patients who agreed to use A3 prosthesis (AK Medical, Beijing, China). However, both the candidates whose bones from bony resection could not be obtained integrally during TKA and those who were added extra bone cuts after PSI-assisted bony resection were excluded (Figure 1). We have retrospectively collected data on 42 patients. The cohort consisted of 38 (90.5%) women and 4 (9.5%) men. Mean age was 69.1 years (range: 54-81 years), and mean body mass index (BMI) was 26.4 kg/m^2^ (range: 19.2-35.6 kg/m^2^). All the 42 cases were varus knees. More detailed information is shown in Table 1. The data are anonymous, and the requirement for informed consent was therefore waived. The study protocol was approved by the ethics committee of Peking University International Hospital (YJ2017-020 and 2019-030BMR). All operations were performed in accordance with relevant guidelines and regulations.

### 2.2. Preoperative Planning and PSI Production

**Figure 2:**
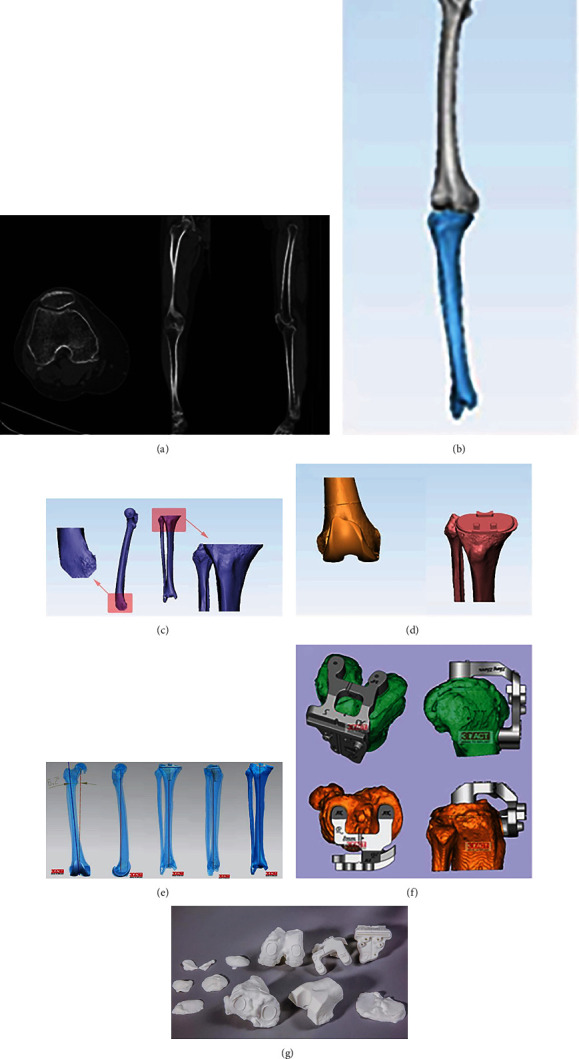
Standard workflow of the preoperation protocol. (a) Full-leg CT scans. (b) Three-dimensional digital reconstructions of the femur and the tibia by UG/NX 10.0 (Siemens PLM Software Ltd., Germany). (c) Simulated bone cutting. (d) Assembling components of the prosthesis and matching them with the bones. (e) Measuring the femur and the tibia, and checking the joint alignment. (f) Designing the PSI according to the virtual surgery. (g) Printing the PSI.

All patients received an A3 posterior-stabilized prosthesis (AK Medical, Beijing, China). Informed consent of receiving TKA by PSI was acquired before the operation for all patients. A computed tomography scan from the pelvis to the foot should be done as soon as a patient decided to accept TKA. Afterward, a full preoperation plan was drafted according to the data from the CT scans, which was followed by a set of three-dimensional- (3D-) printed PSI (Figure 2(g)). From the CT data to the PSI, a constant group consisting of one surgeon and two mechanical engineers would finish a preoperation protocol following a standard workflow (Figure 2). A full preoperation protocol would provide the surgeon with all the critical references needed during a TKA operation, including the bony resection thickness, rotation alignment, frontal alignment, sagittal alignment, and prosthesis sizes. Before an operation, the paper version of the preoperative plan would be printed and hung on the wall in the operation room. During the surgery, the actual situation should be checked. If the PSI could not match the bones, they would be abandoned, and the conventional instruments would be used instead.

### 2.3. Surgical Procedure

**Figure 3:**
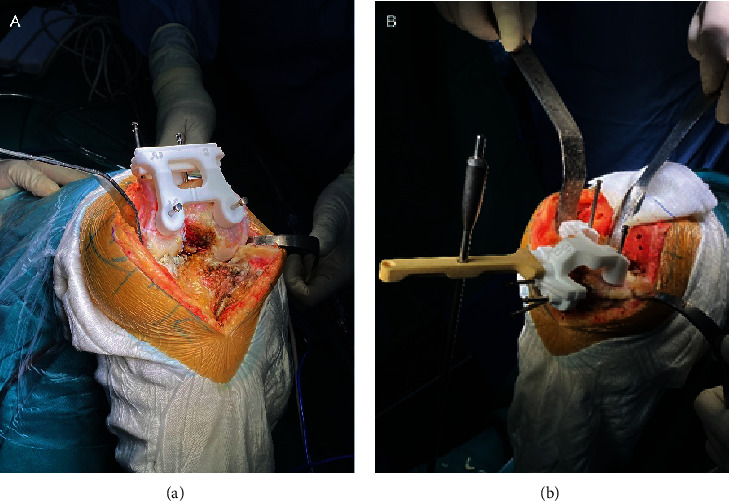
PSI-assisted femur (a) and tibia (b) cutting. PSI were fixed correctly on the bones before cutting.

A medial parapatellar approach was adopted in all TKA surgeries. After appropriate soft tissue release, joint exposure, and scraping of the articular cartilage, PSI components were fixed on the bone surface correctly, and bone cuts were conducted subsequently (Figure 3). Femoral cuts were followed by tibial cuts. The distal femoral condyle and tibial plateau osteotomies were assisted by PSI, while the posterior femoral condyle and anterior femoral condyle osteotomies were performed with conventional jigs. All the bony resections were operated with a 1.27 mm thick saw blade. All the operations were performed by the same group of senior doctors.

### 2.4. Bony Resection Measurement

**Figure 4:**
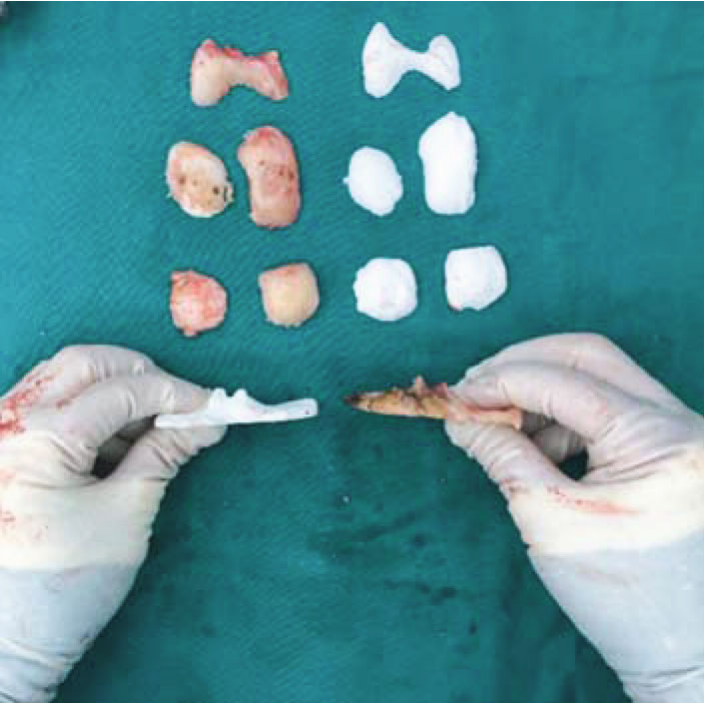
Actual cut bones (the bloody color) and 3D-printed planned cut references (the white color).

**Figure 5:**
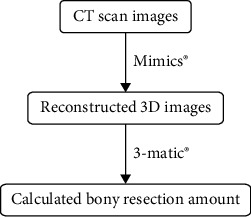
Workflow of the bony resection measurement.

**Figure 6:**
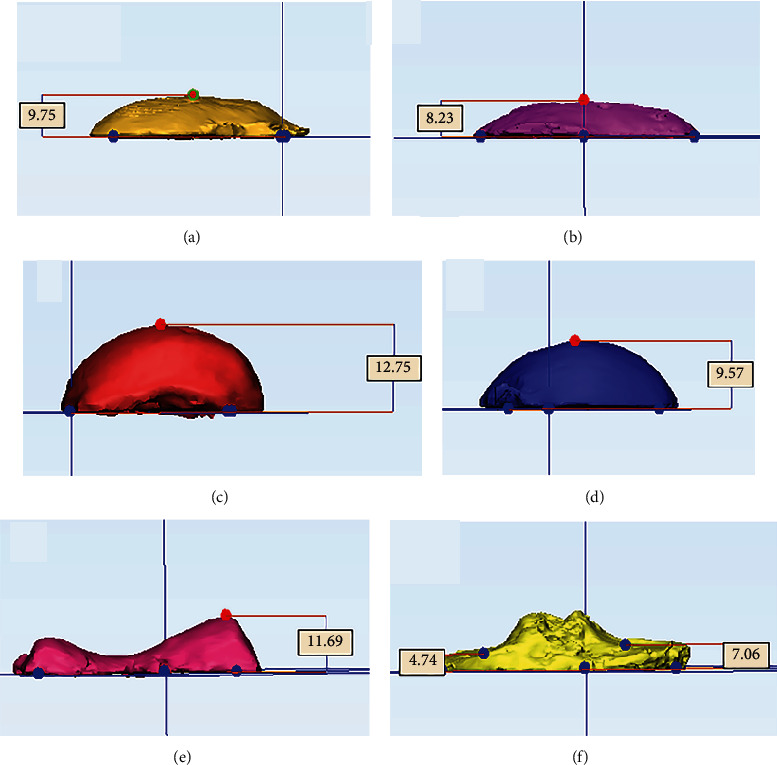
Measurement of the bony resection by 3-matic®. (a) Distal medial femoral condyle. (b) Distal lateral femoral condyle. (c) Posterior medial femoral condyle. (d) Posterior lateral femoral condyle. (e) Anterior femoral condyle. (f) Tibial plateau.

Bone fragments including the distal medial femoral condyle (DMFC), the distal lateral femoral condyle (DLFC), the posterior medial femoral condyle (PMFC), the posterior lateral femoral condyle (PLFC), the anterior femoral condyle (AFC), the medial tibial plateau (MTP), and the lateral tibial plateau (LTP) were collected after the completion of femoral and tibial bony resection. The surgeon would manually compare the bone fragments with the printed models one by one (Figure 4). In the following steps, CT scans were used for the bone fragments to obtain the radiological images. CT data were imported to Mimics® 19.0 (Materialise NV, Belgium), and three-dimensional images were reconstructed. Bony resection amount was calculated by 3-matic® software (version 11.0, Materialise NV, Belgium) based on 3D images imported from Mimics® (Figures 5 and 6). All the measurements were done by a doctor (Liang Yuan) blind to the preoperative surgical plans. The difference of osteotomy was obtained by subtracting the planned osteotomy amount from the actual osteotomy amount. Negative numbers indicate inadequate osteotomy (undercut), while positive numbers mean excessive osteotomy (overcut). The magnitude of the difference is measured in absolute value.

### 2.5. Statistical Analysis

Categorical variables were presented as frequencies and percentages. Continuous variables were summarised as either means and standard deviations or medians with interquartile ranges. To find out potential patterns of error, the proportion of outliers was compared between femoral and tibial cuts, and a Chi-square test was performed. All analyses were performed with the statistical software packages R (http://www.R-project.org, The R Foundation) and EmpowerStats (http://www.empowerstats.com, X&Y Solutions, Inc., Boston, MA).

## 3. Results

### 3.1. Characteristics of the Subjects

A total of 56 patients were included in the study, and 14 cases were excluded because of incomplete bone fragments. Forty-two cases were eventually included in the data analysis. Table 1 shows the basic characteristics of the subjects. The majority of the cases were female patients (38 (90.5%)) with knee osteoarthritis (39 (92.9%)), and their ages were relatively old (69.1 ± 6.7 years). All cases had mild to moderate genu varus deformity (median angle: 9.0 degrees).

### 3.2. The Differences between Actual and Planned Osteotomies

**Figure 7:**
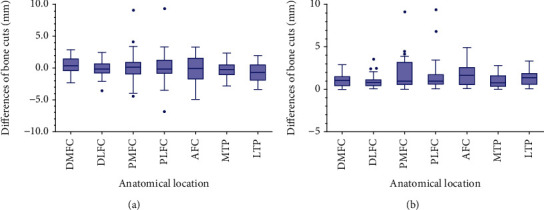
Differences in osteotomy at each anatomical site. (a) The difference of osteotomy was calculated by subtracting the planned osteotomy amount from the actual osteotomy amount. Negative numbers indicate inadequate osteotomy (undercut), while positive numbers mean excessive osteotomy (overcut). (b) The magnitude of the difference is measured in absolute value. Abbreviations: DMFC=distal medial femoral condyle; DLFC=distal lateral femoral condyle; PMFC=posterior medial femoral condyle; PLFC=posterior lateral femoral condyle; AFC=anterior femoral condyle; MTP=medial tibial plateau; LTP=lateral tibial plateau.

**Figure 8:**
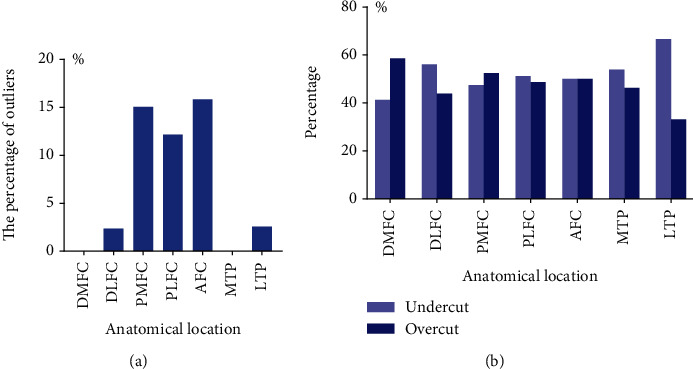
The percentages of outliers (a), and the percentages of undercut and overcut (b) at each anatomical location.

Differences in osteotomy at different anatomical sites were presented in Table 2 and in Figures 7 and 8. The distal femoral condyle had the most accurate bone cuts with the smallest differences (median: 1.0 mm at DMFC and 0.8 mm at DLFC) and the least outliers (none at DMFC and 1 (2.4%) at DLFC). The tibial plateau came in second (median difference: 0.8 mm at MTP and 1.4 mm at LTP; outliers: none at MTP and 1 (2.6%) at LTP). To further compare the distal femur and tibial plateau, DMFC and DLFC and MTP and LTP were summed. Regardless of whether the threshold was set to >2 mm (14 (17.9%) at the tibial plateau vs. 12 (14.6%) at the distal femoral condyle, *p* > 0.05) or >3 mm (1 (1.3%) at the tibial plateau vs. 1 (1.2%) at the distal femoral condyle, *p* > 0.05), the accuracy of tibial plateau osteotomy was similar to that of the distal femoral condyle. Osteotomy accuracy at the posterior femoral condyle and the anterior femoral condyle was the worst. Outliers were up to 6 (15.0%) at PMFC, 5 (12.2%) at PLFC, and 6 (15.8%) at AFC. The percentages of overcut and undercut tended to be 50% in most parts except LTP. At LTP, the undercut percentage was twice that of overcut.

## 4. Discussion

This case series study mainly describes the accuracy of PSI in replicating preoperatively programmed osteotomy with the usage of the CT-based measuring method. With the assistance of PSI, the distal femoral condyle and tibial plateau osteotomies showed small differences with the preoperative plan. This implied that PSI might have good accuracy in bone cuts during TKA.

To our best knowledge, this is the first study to use CT data to measure the bony resection amount. Previous similar studies used the vernier caliper [18, 21, 22]. It is well known that PSI are patient-specific cutting guides based on CT or MRI images, and that preoperatively planned bone cuttings are also measured from CT or MRI data [10, 16, 23]. We assume that the error of the micrometer measurement may be greater than that of the CT or MRI data. In this study, both the preoperative and postoperative measurements were based on CT data. In theory, measurements with the same method can result in much smaller errors. In addition, the measurement based on CT data can effectively avoid the influence of the cartilage layer. Clinical experience tells us that more or less cartilage remained on the surface of the bones. When a vernier caliper was used, the measured value must have included the cartilage layer thickness, which would bias the research results.

Several previous studies have explored the accuracy of PSI-assisted osteotomy [19, 21, 22, 24–26]. All of them reported high accuracy of distal femoral condyle osteotomy. Zambianchi et al. [24] found that the proportion of differences within ±2 mm between intraoperative measured bony resections and planned bone cuts occurred in more than 90% of the cohort for distal femoral resections. Nankivell et al. [21] and Kievit et al. [26] reported even better accuracy, which showed that average resection errors were less than 1 mm both medially and laterally for the distal femur. However, the accuracy of the tibial plateau osteotomy did not seem to be so good. The percentage of osteotomy error within ±2 mm on the proximal lateral tibia was less than 70%, according to Okada et al. [25]. Levy et al. [22] also reported a similar result, which showed that the tibial cuts had the lowest proportion of acceptable cuts (68.9%). In this study, we got different results. Regardless of whether the threshold was set to >2 mm (14 (17.9%) at the tibial plateau vs. 12 (14.6%) at the distal femoral condyle, *p* > 0.05) or >3 mm (1 (1.3%) at the tibial plateau vs. 1 (1.2%) at the distal femoral condyle, *p* > 0.05), the accuracy of tibial plateau osteotomy was similar to that of the distal femoral condyle.

In this study, we found that osteotomy tended to be undercut at LTP. Generally, the percentages of overcut and undercut were close to 50% in most parts except LTP. At LTP, the undercut percentage was twice that of overcut (Figure 8(b)). This may be attributed to the anatomical characteristics of the tibial plateau. The medial side of the tibial plateau was concave, and the lateral side was convex, and as a consequence, the attachment point of the PSI on the outside was easier to shift. This phenomenon was indeed observed during the surgery. In addition, there was another possible reason. This cohort entirely consisted of varus knees, which were characterized by more wear of the medial cartilage [27]. Therefore, a thicker cartilage layer remained on the lateral side. If the lateral cartilage was not removed cleanly before the PSI attached, it would easily lead to less osteotomy. This finding reminds us to pay special attention to the pitfalls of PSI-assisted TKA caused by the difference between the medial and lateral sides of the tibial plateau during the operation.

Besides, there was another interesting finding in this study. Compared with the distal femoral condyle and tibial plateau, there were much more outliers at PMFC (6 (15.0%)), PLFC (5 (12.2%)), and AFC (6 (15.8%)). This implies that the accuracy of osteotomy of the posterior femoral condyle and anterior femoral condyle was much worse. Coincidentally, the posterior femoral condyle osteotomy and the anterior femoral condyle osteotomy were performed with traditional metal jigs rather than the 3D-printed PSI. This suggested that PSI accuracy might be better than traditional tools. Nevertheless, this still needs to be confirmed by a later study that we are going to conduct next.

This study also has several limitations. Firstly, the majority of the cases in this study were female patients. Secondly, all cases were genu varus. Therefore, the data in this study cannot describe whether valgus knees have similar results. We assume that valgus knees are likely to have different results, and one previous study also suggested this [22]. Moreover, given that this was a descriptive study with no controls, it was not able to determine whether PSI would be superior to conventional tools in bone cuttings, although some of the results suggested that possibility. In the end, we should be very cautious in interpreting the results of this article. The purpose of this study was to describe the accuracy of the bony resection with PSI in transferring the preoperative plan to TKA surgery. The concept of PSI accuracy cannot be easily extended to problems related to lower limb alignment. Numerous factors may affect the lower limb alignment even if a precise osteotomy is performed.

## 5. Conclusion

The tibial plateau and distal femoral condyle share a similar accuracy of osteotomy with PSI. PSI have a generally good accuracy during the femur and tibia bone resection in TKA. PSI could be a kind of user-friendly tool which can simplify TKA with good accuracy.

## Figures and Tables

**Table 1:** Baseline characteristics of participants.

Characteristics	All patients (*n* = 42)
Sex	
Male, *n* (%)	4 (9.5%)
Female, *n* (%)	38 (90.5%)
Age (years, mean ± SD)	69.1 ± 6.7
Height (meter, mean ± SD)	1.6 ± 0.1
Weight (kg, mean ± SD)	66.7 ± 10.2
BMI (mean ± SD)	26.4 ± 3.4
Extension (degree, median, Q1-Q3)	8.0 (3.0-15.0)
Flexion (degree, mean ± SD)	113.0 ± 15.3
HKA (degree, mean ± SD)	169.9 ± 5.5
Alignment angle (degree, median, Q1-Q3)	9.0 (6.2-12.8)
Side	
Left, *n* (%)	22 (52.4%)
Right, *n* (%)	20 (47.6%)
Diagnosis	
OA, *n* (%)	39 (92.9%)
RA, *n* (%)	3 (7.1%)
Alignment	
Varus, *n* (%)	42 (100.0%)
Valgus, *n* (%)	0

Abbreviations: BMI=body mass index; HKA=hip-knee-ankle angle; OA=osteoarthritis; RA=rheumatoid arthritis.

**Table 2:** Differences between actual bone cuts and surgical plan at each location^a^.

Anatomical location	Median, Q1-Q3 (mm)	≤1.0mm, *n* (%)	1.1-2.0 mm, *n* (%)	2.1-3.0mm *n* (%)	Outliers,^b^*n* (%)	Undercut *n* (%)	Overcut, *n* (%)
DMFC	1.0 (0.4-1.5)	20 (48.8%)	14 (34.1%)	7 (17.1%)	0	17 (41.5%)	24 (58.5%)
DLFC	0.8 (0.4-1.0)	28 (68.3%)	8 (19.5%)	4 (9.8%)	1 (2.4%)	23 (56.1%)	18 (43.9%)
PMFC	1.0 (0.5-1.9)	21 (52.5%)	11 (27.5%)	2 (5.0%)	6 (15.0%)	19 (47.5%)	21 (52.5%)
PLFC	1.0 (0.6-1.7)	21 (51.2%)	12 (29.3%)	3 (7.3%)	5 (12.2%)	21 (51.2%)	20 (48.8%)
AFC	1.6 (0.5-2.6)	13 (34.2%)	9 (23.7%)	10 (26.3%)	6 (15.8%)	19 (50.0%)	19 (50.0%)
MTP	0.8 (0.3-1.6)	22 (56.4%)	12 (30.8%)	5 (12.8%)	0	21 (53.8%)	18 (46.2%)
LTP	1.4 (0.6-1.9)	18 (46.2%)	12 (30.8%)	8 (20.5%)	1 (2.6%)	26 (66.7%)	13 (33.3%)

^a^All the numbers in the table were calculated by absolute value except the undercut and overcut columns. ^b^The outliers were defined as >3mm. Abbreviations: DMFC=distal medial femoral condyle; DLFC=distal lateral femoral condyle; PMFC=posterior medial femoral condyle; PLFC=posterior lateral femoral condyle; AFC=anterior femoral condyle; MTP=medial tibial plateau; LTP=lateral tibial plateau.

## Data Availability

The data supporting the results are available from the corresponding authors upon request.
